# On the effect of hippocampal c-Jun N-terminal kinase inhibition on object recognition memory

**DOI:** 10.3389/fnbeh.2022.1052124

**Published:** 2022-12-12

**Authors:** Janine I. Rossato, Andressa Radiske, Maria Carolina Gonzalez, Lia R. M. Bevilaqua, Martín Cammarota

**Affiliations:** ^1^Memory Research Laboratory, Brain Institute, Federal University of Rio Grande do Norte, Natal, Rio Grande do Norte, Brazil; ^2^Department of Physiology, Federal University of Rio Grande do Norte, Natal, Rio Grande do Norte, Brazil; ^3^Edmond and Lily Safra International Institute of Neuroscience, Macaíba, Rio Grande do Norte, Brazil

**Keywords:** consolidation, reconsolidation, recall, amnesia, hippocampus, SP600125

## Abstract

c-Jun N-terminal kinase (JNK) phosphorylates the transcription factor c-Jun in response to stress stimuli and contributes to both hippocampal synaptic plasticity and memory processing in mammals. Object recognition memory (ORM) is essential for remembering facts and events. In rodents, ORM consolidation and reconsolidation require a functional hippocampus. However, the possible involvement of hippocampal JNK on ORM processing has not yet been studied. Here we show that when injected into dorsal CA1 5 min, but not 6 h, after training adult male rats in the novel object recognition learning task, the JNK inhibitor SP600125 impaired ORM for at least 7 days without affecting exploratory activity, short-term ORM retention, or the functional integrity of the hippocampus. SP600125 did not hinder ORM retention when given in CA1 after a memory reactivation session carried out 24 h post-training in the presence of the same two objects presented during the training session, but caused time-dependent amnesia when one of the objects presented at training was replaced by a different but behaviorally equivalent novel one. Taken together, our results indicate that hippocampal JNK activity is necessary for ORM consolidation and reconsolidation but not for ORM recall or short-term retention.

## Introduction

c-Jun N-terminal kinases (JNKs) are a group of 46–55 kDa stress-responsive protein kinases encoded by the JNK1, JNK2, and JNK3 genes that belong to the mitogen-activated protein kinase family. Originally identified as the kinase activity that phosphorylates the transcription factor c-Jun, it is now clear that JNK also couples cytokines- and growth factors-signaling to other nuclear and non-nuclear effectors, including the transcription factors ATF2, STAT3, and ELK1, the adaptor protein paxillin, the mitochondrial membrane protein BCL-2, and the protein kinases Akt and p90RSK, to regulate cell growth, differentiation, and apoptosis. In the brain, aside from its well-described participation in axodendritic morphogenesis (Komulainen et al., 2020), JNK signaling influences the pathogenesis of Alzheimer’s disease (AD; Yarza et al., 2016), a progressive neurodegenerative illness that results in the loss of cognitive functioning. In fact, JNK seems to play important roles in synaptic plasticity and non-declarative memory. In this respect, mutant mice expressing an unphosphorylable c-Jun isoform show impaired hippocampal long-term potentiation (LTP; Seo et al., 2012), whereas pharmacological inhibition of hippocampal JNK enhances short-term memory and paired pulse facilitation and rescues stress-induced contextual fear conditioning from amnesia but blocks long-term fear-motivated avoidance memory consolidation, recall, and extinction (Bevilaqua et al., 2003, 2007; Li et al., 2007; Sherrin et al., 2010). However, it is currently unknown whether JNK is also involved in episodic memory, the type of declarative memory affected early in AD (Bäckman et al., 2001). Object recognition memory (ORM) allows animals to identify familiar items and is essential for remembering episodic information (Cole et al., 2019). In rats, ORM consolidation requires the functional integrity of several brain structures (Rossato et al., 2013), including the hippocampus (Clarke et al., 2010; Furini et al., 2010; ILL-Raga et al., 2013). The hippocampus also participates in ORM reconsolidation, a protein synthesis-dependent process that restabilizes and updates consolidated ORMs destabilized when recalled in the presence of a novel object (Rossato et al., 2007; Radiske et al., 2017; Gonzalez et al., 2021, 2022). Here, we analyzed whether hippocampal JNK is necessary for ORM consolidation and reconsolidation by assessing the effect on retention of the intra-dorsal CA1 administration of SP600125, a potent, cell-permeable, selective, and reversible ATP-competitive inhibitor of JNK (Bennett et al., 2001; Ennis et al., 2005) that does not affect other kinases or signaling pathways presently known to be important for the consolidation, recall, or reconsolidation of ORM in rats.

## Materials and methods

### Subjects

All experiments were performed during the light phase of the daylight cycle in agreement with the National Institutes of Health for the Care and Use of Laboratory Animals and the local institutional ethics committee [Comissão de Ética no Uso de Animais (CEUA) and UFRN] recommendations. We used a total of 198 adult male Wistar rats (3 months old; 300–350 g). They were housed in groups of five per cage and kept at 23°C in the institutional vivarium on a 12 h lights on/off schedule (lights on at 6:00 a.m.) with *ad libitum* access to food and water.

### Stereotaxic surgery

Rats were anesthetized with ketamine (80 mg/kg)/xylazine (10 mg/kg) and bilaterally implanted with 22-gauge stainless steel cannula guides aimed to the CA1 region of the dorsal hippocampus (AP −4.2; LL, ±3.0; DV, −3.0). Stereotaxic coordinates were taken from Paxinos and Watson (2007). Rats received meloxicam (0.2 mg/kg) at the end of the surgical procedures and were allowed to recover for 7 days.

### Drugs and injection procedures

SP600125 was obtained from Sigma-Aldrich (São Paulo, Brazil), dissolved in DMSO upon arrival, aliquoted, stored at −20°C and diluted to working concentration in sterile saline (0.9%) on the day of the experiment. For drug delivery, injection cannulas were fitted into the guides and injections (1 μl/side at 0.5 μl/min) carried out using a Hamilton syringe coupled to an infusion pump. The injection cannulas were left in place for 1 minute to minimize backflow. An equal volume of 0.1% DMSO in sterile saline was used as vehicle (VEH) control.

### Novel object recognition task

Novel object recognition training and testing was conducted in a gray plywood open-field arena (60 cm × 60 cm × 60 cm) placed in a dim-light illuminated room acclimatized at 23–24°C, as described (Myskiw et al., 2008; Rossato et al., 2015). Briefly, rats were handled and allowed to explore the training arena in the absence of objects for 20 min/day during 4 days (habituation sessions). Twenty-four hours after the last habituation session, rats were exposed to two identical copies of the same novel object (object A) for 5 min in the training arena to induce ORM formation. To reactivate ORM, 24 h after training animals were re-exposed to familiar object A alongside novel object B in the training arena for 5 min. ORM retention was assessed only once per animal in a test session carried out 3 h, 24 h, or 7 days after training or reactivation. During the retention test, rats were exposed to familiar object A along with novel object C for 5 min. One hour before the experimental sessions, rats were transported from the vivarium to the experimental anteroom. From there, each rat was individually brought to the experiment room in a transport cage. At the end of each session, rats were returned to the experimental anteroom where they stayed for one additional hour before being transferred back to the vivarium. Objects were made of metal, glass, or glazed ceramic and had no significance for the rats, which showed no innate preference for any of them (Table 1). The open-field arena and the objects were cleaned with 50% ethanol before each trial to ensure absence of olfactory cues. Object exploration was defined as sniffing and touching the objects with the muzzle and/or forepaws. Sitting on or turning around the objects was not considered exploratory behavior. A digital video camera fixed above the open-field arena was used for tracking the position and behavior of the rats. Video data were acquired at 30 frames/s and analyzed using the ObjectScan system (CleverSys). The discrimination index (DI) was calculated as follows: (time exploring novel object–time exploring familiar object)/total object exploration time, considering data from the 5 min session (Rossato et al., 2013). Naive rats discriminated between novel and familiar objects throughout the retention test session (Table 2). DI varied between −1 and +1; positive DI scores indicate preference for the novel object, whereas DI scores close to zero suggests absence of discrimination. Animals were excluded from data analysis when total exploration time during training, reactivation, or test sessions was less than 20 s (3 animals). We also excluded two animals that did not show object preference during reactivation session (RA).

**TABLE 1:** Naive adult male Wistar rats display no innate preference for any of the objects utilized in the novel object recognition (NOR) task.

	Object exploration time (s)					
Object pair	Object 1	Object 2	Total	DI	*p*	*n*
A–A	30.98 ± 3.86	27.17 ± 3.21	58.15 ± 6.24	–0.06	0.264	11
A–B	28.58 ± 3.33	29.00 ± 3.86	57.58 ± 6.56	0.002	0.971	10
A–C	24.74 ± 2.50	24.19 ± 3.59	49.64 ± 7.44	–0.03	0.706	10
B–C	24.74 ± 2.50	26.33 ± 4.06	51.07 ± 6.00	–0.02	0.780	10

The table shows mean exploration time and DI ± SEM for naive animals during spontaneous object exploration in the training session of the NOR task. Total exploration time did not differ between objects pairs [*F*(3,37) = 0.445, *p* = 0.7223]. Discrimination indexes (DIs) are shown. *p* in one-sample Student’s *t* test with theoretical mean = 0.

**TABLE 2:** Adult male Wistar rats trained in the novel object recognition (NOR) task discriminate between novel and familiar objects throughout the entire retention test session.

	1st min	2nd min	3rd min	4th min	5th min
DI	0.19 ± 0.08	0.20 ± 0.07	0.23 ± 0.04	0.20 ± 0.06	0.21 ± 0.08
*p*	0.0411	0.0167	0.0001	0.0112	0.0275
Object exploration time (s)	18.80 ± 2.18	16.27 ± 1.96	15.55 ± 2.03	15.51 ± 1.75	14.27 ± 1.49

The table shows mean ± SEM, discrimination index (DI), and total exploration time for each consecutive minute of a 5-min-long object recognition memory (ORM) retention test session in the presence of familiar object A and novel object C performed 24 h after NOR training in the presence of two identical novel objects A. *p* in one-sample Student’s *t* test with theoretical mean = 0 (*n* = 11).

### Step-down inhibitory avoidance task

Inhibitory avoidance training was carried out as previously described (Rossato et al., 2006; Radiske et al., 2015). The IA training chamber was made of Plexiglas (50 cm × 25 cm × 25 cm) and contained an elevated wooden platform (5 cm × 8 cm × 25 cm) positioned at its left end. The floor of the chamber was a grid of bronze bars connected to a shock generator. At the beginning of the training session, animals were placed on the wooden platform and received a scrambled footshock (0.4 mA for 2 s) immediately after they stepped down to the grid. IA memory retention was evaluated 24 h after training by placing the animals on the training chamber platform and measuring their latency to step down. The test session finished when the animals stepped down to the grid or after 300 s, whatever happened first.

### Data analysis

Statistical analyses were performed using GraphPad Prism 8 software. Significance was set at *p* < 0.05. NOR data were analyzed using one-sample *t* test with theoretical mean = 0 or two-way ANOVA followed by Bonferroni’s multiple comparisons, as appropriate. IA data were analyzed using Mann–Whitney *U* test.

## Results

**FIGURE 1:**
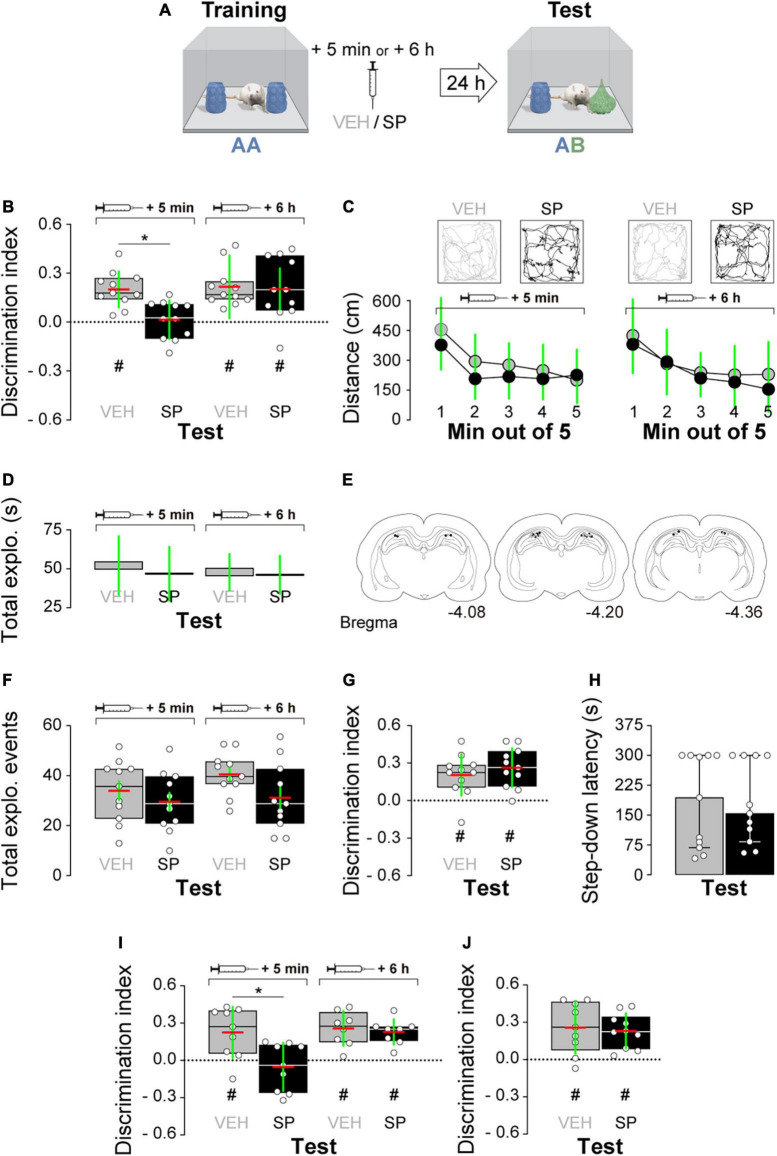
**(A)** Experimental protocol. **(B)** Rats trained in the novel object recognition (NOR) task using two copies of object A received bilateral intra-dorsal CA1 injections of SP600125 (SP; 20 μM; 1 μl/side) or vehicle (VEH; 0.1% DMSO in sterile saline) 5 min or 6 h post-training. Object A memory retention was assessed 24 h later (test) in the presence of familiar object A and novel object B. **(C**, top**)** Representative trajectory for VEH and SP-treated animals during Test. **(C**, bottom**)** Mean distance traveled during test for VEH and SP-treated animals. **(D)** Total object exploration time during Test. **(E)** Illustration showing cannula placement for animals that received VEH or SP 5 min after training. **(F)** Number of exploration events during Test. **(G)** Animals that received SP or VEH 5 min after training were retrained in NOR using a different pair of novel stimuli objects. Object recognition memory (ORM) retention was evaluated 24 h thereafter. **(H)** Animals that received SP or VEH 5 min after NOR training were trained in the inhibitory avoidance (IA) task. IA memory retention was evaluated 24 h thereafter. **(I)** Rats were treated as in panel **(A)** except that the retention test was performed 7 days post-training. **(J)** Rats were treated as in panel **(A)** except that the retention test was performed 3 h post-training. Discrimination index (DI) data are expressed as median (black or white horizontal lines) ± interquartile range (boxplots) and as mean (red horizontal line) ± SD (green vertical line). Dashed lines represent chance level. Total object exploration and distance traveled data are presented as mean ± SD. IA data are expressed as median ± interquartile range. *n* = 8–12 animals per group; ^#^*p* < 0.05 in one-sample Student’s *t*-test with theoretical mean = 0; and *p < 0.05 in Bonferroni’s multiple-comparison test after two-way ANOVA.

**FIGURE 2:**
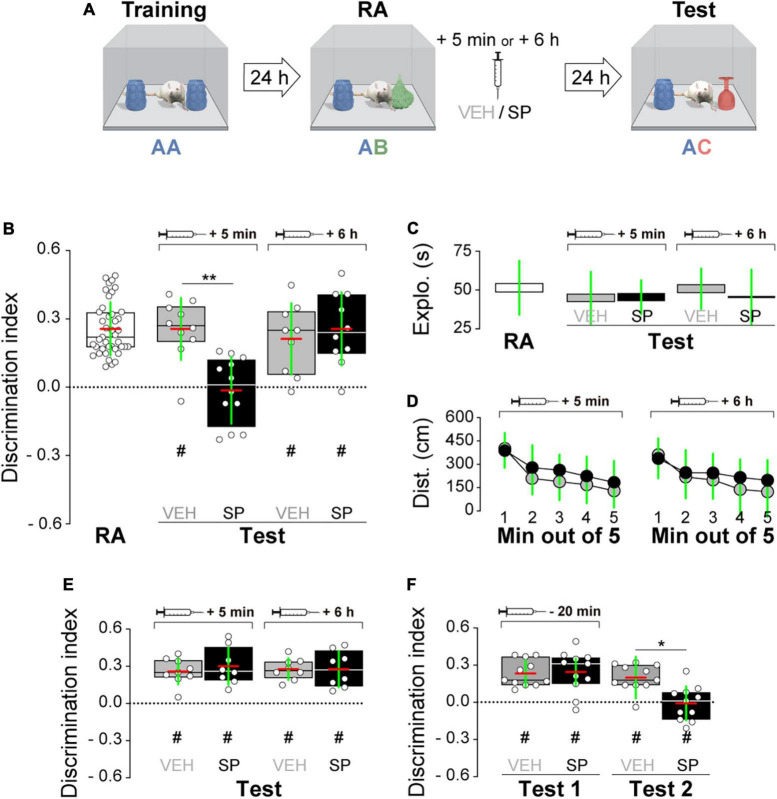
**(A)** Experimental protocol. **(B)** Rats were trained in the novel object recognition (NOR) task using two copies of object A and 24 h later they were submitted to an object recognition memory (ORM) reactivation session (RA) in the presence of familiar object A and novel object B. Five min or 6 h after RA, rats received bilateral intra-dorsal CA1 injections of SP600125 (SP; 20 μM; 1 μl/side) or vehicle (VEH; (0.1% DMSO in sterile saline). One day later rats were exposed to familiar object A and novel object C to evaluate ORM retention (Test). **(C)** Total exploration time during test. **(D)** Mean distance traveled during test for VEH and SP-treated animals. **(E)** Rats were treated as in panel **(A)**, except that RA occurred in the presence of two copies of familiar object A. **(F)** Rats were treated as in panel **(A)**, except that the animals received bilateral intra-CA1 injections of VEH or SP 20 min before Test 1. Discrimination index (DI) data are expressed as median (black or white horizontal lines) ± interquartile range (boxplots) and as mean (red horizontal line) ± SD (green vertical line). Dashed lines represent chance level. Total object exploration and distance traveled data are presented as mean ± SD. *n* = 8–12 animals per group; ^#^*p* < 0.05 in one-sample Student’s *t*-test with theoretical mean = 0; and *p < 0.05 and **p < 0.01 in Bonferroni’s multiple-comparison test after two-way ANOVA.

Firstly, we examined whether hippocampal JNK inhibition affects ORM consolidation. To do that, we implanted adult male Wistar rats with guide cannulas aimed to the CA1 region of the dorsal hippocampus and trained them in the NOR task, an incidental episodic-like learning paradigm based on the rodents’ innate preference for novelty (Ennaceur and Delacour, 1988; Clarke et al., 2008) involving exposure to two identical novel stimuli objects A in a familiar open field arena. Five minutes or 6 h after training, animals received bilateral intra-dorsal CA1 injections (1 μl) of VEH (0.1% DMSO in sterile saline) or the JNK inhibitor SP600125 (20 μM; Bevilaqua et al., 2003, 2007), and 24 h post-training were exposed to one copy of familiar object A alongside a novel object B for 5 min to evaluate object A memory retention (Figure 1A). As can be seen in Figure 1B, animals that were given VEH discriminated novel object B from familiar object A during the retention test session regardless of the time elapsed between the training session and the moment of the injections. However, animals that received SP600125 5 min after training, but not 6 h thereafter, were unable to discriminate between objects A and B [Figure 1B; *F*(1,40) = 5.802, *p* = 0.0207 for treatment; *F*(1,40) = 5.598, *p* = 0.0229 for injection time, and *F*(1,40) = 4.251, *p* = 0.0458 for interaction; *t*(40) = 3.131, *p* < 0.05 for VEH 5 min vs. SP 5 min, *t*(40) = 3.376, *p* < 0.01 for VEH 6 h vs. SP 5 min, and *t*(40) = 3.161, *p* < 0.05 for SP 5 min vs. SP 6 h in Bonferroni’s multiple comparisons test after two-way ANOVA]. SP600125 did not affect total distance traveled (Figure 1C), total exploration time (Figure 1D), or the total number of exploration events during the test session (Figure 1F). See Figure 1E for an illustration showing the position of injection cannulas in animals that received VEH or SP600125 5 min after training. Rats rendered amnestic with SP600125 were able to acquire and recall ORM upon retraining (Figure 1G) as well as to learn and express a fear-motivated avoidance response (Figure 1H) when trained in a step-down IA task (Alonso et al., 2005; Kerr et al., 2005; Bekinschtein et al., 2007), which also requires the functional integrity of the hippocampal formation (Bernabeu et al., 1995; Cammarota et al., 1998; Paratcha et al., 2000; da Silva et al., 2006; Katche et al., 2010). The amnesia caused by SP600125 lasted for at least 7 days [Figure 1I; *F*(1,30) = 7.54, *p* = 0.0101 for treatment, *F*(1,30) = 7.235, *p* = 0.0116 for injection time, and *F*(1,30) = 4.871, *p* = 0.0351 for interaction; *t*(30) = 3.569, *p* < 0.01 for VEH 5 min vs. SP 5 min, *t*(30) = 3.844, *p* < 0.01 for VEH 6 h vs. SP 5 min, and *t*(30) = 3.502, *p* < 0.01 for SP 5 min vs. SP 6 h in Bonferroni’s multiple comparisons test after two-way ANOVA], but was not observed when ORM retention was assessed 3 h post-training (Figure 1J). The hippocampus is engaged in ORM reconsolidation in the NOR task only when the memory of the familiar object is reactivated in the presence of a novel one (Gonzalez et al., 2019; Rossato et al., 2019). Therefore, to analyze the possible participation of hippocampal JNK on ORM reconsolidation, 24 h post-training NOR-trained rats were re-exposed for 5 min to one copy of familiar object A alongside novel object B to reactivate the memory for object A and induce its hippocampus-dependent reconsolidation. Five minutes post-reactivation, or 6 h thereafter, animals received bilateral intra-CA1 injections of VEH or SP600125 (20 μM). Retention of the memory for object A was assessed 24 h afterward by exposing the animals to one copy of this object alongside novel object C (Figure 2A). Rats that received VEH or SP600125 6 h after object A memory reactivation discriminated this object from object C during the retention test session; animals that were given VEH 5 min after object A memory reactivation also remembered it 24 h later, but those given SP600125 failed to do so [Figure 2B; *F*(1,37) = 4.573, *p* = 0.00391 for treatment, *F*(1,37) = 4.573, *p* = 0.0391 for injection time, and *F*(1,37) = 9.465, *p* = 0.0039 for interaction; *t*(37) = 3.825, *p* < 0.01 for VEH 5 min vs. SP 5 min, *t*(37) = 3.046, *p* < 0.05 for VEH 6 h vs. SP 5 min, and *t*(37) = 3.825, *p* < 0.01 for SP 5 min vs. SP 6 h in Bonferroni’s multiple comparisons test after two-way ANOVA]. Post-reactivation intra-CA1 SP600125 administration did not affect total exploration time (Figure 2C), or total distance traveled (Figure 2D) during the test session. As expected, neither VEH nor SP600125 had any effect on retention when injected in dorsal CA1 5 min or 6 h after submitting animals to an ORM RA in the presence of two copies of object A (Figure 2E). Pre-test intra-CA1 SP600125 administration did not impair ORM recall, but hampered object A memory retention during a second test session carried out 24 h after the first one in the presence of object A and novel object C [Figure 2F; *F*(1,40) = 11.60, *p* = 0.0015 for treatment, *F*(1,40) = 4.912, *p* = 0.0324 for injection time, and *F*(1,40) = 6.327, *p* = 0.016 for interaction; VEH-Test 1 vs. SP-Test 2: *t*(40) = 3.975, *p* < 0.01, VEH-Test 2 vs. SP-Test 2: *t*(40) = 3.346, *p* < 0.05, and SP-Test 1 vs. SP-Test 2: *t*(40) = 4.187, *p* < 0.001 in Bonferroni’s multiple comparisons test after two-way ANOVA]. Table 3 shows statistics for control experiments.

**TABLE 3:** Detailed statistics for control experiments.

Figures	Statistical method	*n*	Statistical details	
1C	Two-way ANOVA for infusion 5 min post-training	VEH: *n* = 11 SP: *n* = 11	Time vs. treatment: *F*(4,80) = 1.49 Treatment: *F*(1,20) = 2.258	*P* = 0.2132 *P* = 0.1485
	Two-way ANOVA for infusion 6 h post-training	VEH: *n* = 11 SP: *n* = 11	Time vs. treatment: *F*(4,80) = 0.8701 Treatment: *F*(1,20) = 0.8256	*P* = 0.4857 *P* = 0.3744
1D	Two-way ANOVA	VEH 5 min: *n* = 11 SP 5 min: *n* = 11 VEH 6 h: *n* = 11 SP 6 h: *n* = 11	Interaction: *F*(1,40) = 0.1265 Infusion time: *F*(1,40) = 0.5847 Treatment: *F*(1,40) = 0.2946	*P* = 0.7240 *P* = 0.449 *P* = 0.5903
1F	Two-way ANOVA	VEH 5 min: *n* = 11 SP 5 min: *n* = 11 VEH 6 h: *n* = 11 SP 6 h: *n* = 11	Interaction: *F*(1,40) = 0.5205 Infusion time: *F*(1,40) = 3.822 Treatment: *F*(1,40) = 1.399	*P* = 0.4748 *P* = 0.0576 *P* = 0.2439
1G	Unpaired *t* test	VEH: *n* = 11 SP: *n* = 11	*t*(20) = 0.8229	*P* = 0.4203
1H	Mann–Whitney test	VEH: *n* = 11 SP: *n* = 11	*U* = 57	*P* = 0.8327
1J	Unpaired *t* test	VEH: *n* = 9 SP: *n* = 10	*t*(17) = 0.3729	*P* = 0.7138
2C	Two-way ANOVA	VEH 5 min: *n* = 10 SP 5 min: *n* = 12 VEH 6 h: *n* = 9 SP 6 h: *n* = 10	Interaction: *F*(1,37) = 0.3876 Infusion time: *F*(1,37) = 0.2829 Treatment: *F*(1,37) = 0.4121	*P* = 0.5374 *P* = 0.5980 *P* = 0.5249
2D	Two-way ANOVA for infusion 5 min post-training	VEH: *n* = 10 SP: *n* = 12	Time vs. treatment *F*(4,80) = 1.494 Treatment F(1,20) = 2.602	*P* = 0.2119 *P* = 0.1224
	Two-way ANOVA for infusion 6 h post-training	VEH: *n* = 9 SP: *n* = 10	Time vs. treatment *F*(4,68) = 1.096 Treatment *F*(1,17) = 1.95	*P* = 0.3655 *P* = 0.1806
2E	Two-way ANOVA	VEH 5 min: *n* = 9 SP 5 min: *n* = 8 VEH 6 h: *n* = 8 SP 6 h: *n* = 8	Interaction: *F*(1,29) = 0.2131 Infusion time: *F*(1,29) = 0.2395 Treatment: *F*(1,29) = 0.0035	*P* = 0.6478 *P* = 0.6282 *P* = 0.9534

## Discussion

Previously, we showed that ORM consolidation and reconsolidation after recall in the presence of a novel object require *de novo* protein synthesis in the hippocampus (Rossato et al., 2007; Myskiw et al., 2008). Here, we corroborated that the hippocampus is necessary for ORM consolidation, confirmed that ORM reactivation in the presence of a novel object induces hippocampus-dependent reconsolidation, and demonstrated that hippocampal JNK is necessary for these two processes. We also presented evidence showing that short-term ORM does not require JNK activity in dorsal CA1, which is not surprising given that short-term ORM does not appear to involve the hippocampal formation (Cohen et al., 2013). Our results can be unambiguously interpreted as due to the inhibitory action of SP600125 on JNK. Indeed, SP600125 hindered ORM retention when injected into dorsal CA1 5 min, but not 6 h after NOR training or ORM recall in the presence of a novel object, which demonstrates that the amnestic effect of this drug was time-dependent and therefore not due to impairment of hippocampal functionality. This claim is further supported by data showing that pre-test intra CA1 injection of SP600125 did not affect ORM memory recall, which requires the normal functionality of the hippocampal formation (Rossato et al., 2019), but hindered subsequent retention, and that animals rendered amnestic for ORM with SP600125 were later able to acquire and express ORM as well as a fear-motivated hippocampus-dependent IA response. Moreover, our experiments also indicate that the amnesic action of SP600125 cannot be attributed to a delayed effect on performance since this drug did not affect total exploration time, total distance travel, or the total number of exploration events during the retention test. JNK inhibitors have a deleterious effect on the consolidation of different hippocampus-dependent memories, including avoidance and extinction memories (Bevilaqua et al., 2003, 2007). These kinases may contribute to the consolidation process in several ways. They adjust the threshold for the induction of long-term synaptic plasticity, modulate neuronal excitability in a bi-directional manner through phosphorylation of AMPAR, and contribute to dendritic spine morphology and density in the hippocampus (Thomas et al., 2008; Komulainen et al., 2020). Moreover, JNK regulates synaptic transmission by controlling the synaptic levels of PSD-95 and thus, the internalization and reinsertion of AMPAR from and to the postsynaptic membrane (Kim et al., 2007), which are necessary steps for ORM destabilization and reconsolidation, respectively (Rossato et al., 2019). Prior work from our group shows that ORM consolidation and reconsolidation are associated with a late period of synaptic enhancement in the dorsal hippocampus (Clarke et al., 2010), and that gene expression and *de novo* protein synthesis in dorsal CA1 are necessary up to 3 h after training or recall for stabilizing new and updated memories (Rossato et al., 2007, 2015; Radiske et al., 2017). In this regard, several transcription factors required for memory maintenance, such as AP-1 and Egr, are rapidly phosphorylated by JNK (Davis, 2000), and it has been reported that JNK knockdown mice show impaired early-LTP to late-LTP transition (Chen et al., 2005). Thus, it is possible that the amnesic effect of SP600125 on ORM is caused by deficient synaptic plasticity in the hippocampus. Given that consolidation and reconsolidation have differential molecular signatures (Bellfy and Kwapis, 2020), further research will be needed to determine whether the plastic changes underlying the storage of newly formed and updated ORM are regulated by different JNK isoforms, although the results we presented here suggest that, in both cases, the events mediated by this kinase occur no later than 6 h after training or recall, respectively. In the last decade, the use of pharmacological interventions as therapeutic co-adjuvants for the treatment of memory-related anxiety disorders has regained momentum. However, the limited number of mnemonically effective drugs that are safe for human use remains one of the major problems of this approach. In this respect, our results are particularly interesting, because several JNK inhibitors are currently being tested in humans as anticancer, antidepressant, and anxiolytic drugs (Hollos et al., 2018; Wu et al., 2020).

## Data availability statement

The original contributions presented in this study are included in this article/supplementary material, further inquiries can be directed to the corresponding author.

## Ethics statement

This animal study was reviewed and approved by the CEUA–UFRN.

## Author contributions

MC supervised the study. All authors conceived and carried out the experiments, analyzed the data, wrote the manuscript, and approved its final version.
